# Panels and models for accurate prediction of tumor mutation burden in tumor samples

**DOI:** 10.1038/s41698-021-00169-0

**Published:** 2021-04-13

**Authors:** Elizabeth Martínez-Pérez, Miguel Angel Molina-Vila, Cristina Marino-Buslje

**Affiliations:** 1 Bioinformatics Unit, Fundación Instituto Leloir, Buenos Aires, C1405BWE, Avda. Patricias Argentinas 435 C1405BWE, Ciudad Autonoma de Buenos Aires, Argentina; 2 Laboratorio de Oncología/Pangaea Oncology, Hospital Universitario Quirón Dexeus, Barcelona, Spain

**Keywords:** Tumour biomarkers, Cancer immunotherapy

## Abstract

Immune checkpoint blockade (ICB) is becoming standard-of-care in many types of human malignancies, but patient selection is still imperfect. Tumor mutation burden (TMB) is being evaluated as a biomarker for ICB in clinical trials, but most of the sequencing panels used to estimate it are inadequately designed. Here, we present a bioinformatics-based method to select panels and mathematical models for accurate TMB prediction. Our method is based on tumor-specific, forward-step selection of genes, generation of panels using a linear regression algorithm, and rigorous internal and external validation comparing predicted with experimental TMB. As a result, we propose cancer-specific panels for 14 malignancies which can offer reliable, clinically relevant estimates of TMBs. Our work facilitates a better prediction of TMB that can improve the selection of patients for ICB therapy.

## Introduction

Tumor cells escape immune surveillance through a variety of mechanisms, including upregulation of three key immune checkpoint proteins, programmed cell death protein 1 (PD-1), PD-1 ligand (PD-L1), and cytotoxic T-lymphocyte associated protein 4 (CTLA-4)^1^. Treatment with antibodies targeting these proteins, known as immune checkpoint blockade (ICB) therapy, can overcome escaping mechanisms, restoring T cell activity and allowing the adaptive component of the immune system to detect and kill the tumor cells. Clinical trials have shown that ICB therapy improves overall survival (OS) of patients with advanced non-small-cell lung cancer (NSCLC), melanoma, renal cell carcinoma, urothelial cancer, Hodgkin’s lymphoma, and other tumors^2–7^. However, not all metastatic patients respond to ICB therapy and only 15–35% of cases derive a durable clinical benefit^1^. In consequence, predictive biomarkers are needed^8^.

At present, evaluation of PD-L1 protein expression by immunohistochemistry (IHC) in tumor and inflammatory cells is the most widely used biomarker for the selection of cancer patients for ICB therapy. It has received FDA-approval as a companion or complementary test in certain malignancies, including NSCLC or urothelial carcinoma^8^. However, IHC presents a number of drawbacks. Several anti-PD-L1 antibodies and cut-off values are used for different immune checkpoint inhibitors (ICIs) and pathologies^9^, and their concordance is far from being perfect^10–12^. In addition, the predictive power of PD-L1 IHC, although well established, is limited; with only 35–45% of patients responding to ICB therapy in the highest expression group^9,11^. Consequently, it is now generally assumed that PD-L1 testing alone is not sufficient and should be complemented with other markers^8,13^. To this end, transcriptomic and epigenetic signatures^14–16^, the presence of tumor-infiltrating lymphocytes^17^, gut microbiome^18–20^, oncogenic mutations^16^, mismatch repair deficiency^21,22^, and tumor mutation burden (TMB)^23^ are currently under investigation. Among them, TMB is probably the biomarker that has received more attention, being the focus of multiple preclinical and clinical studies.

Tumors with high TMB are likely to generate more neoantigens, which can be displayed on HLA molecules on the surface of the cancer cells and be recognized by the immune system^24–27^. A significant number of retrospective studies, some of them performed in tumor samples from clinical trials, have found an association between TMB and outcome to ICIs^23,28–30^. However, some negative results have also been reported^31,32^ and recent clinical trials have yielded contradictory results^33^. Some reasons can help to explain these inconsistencies. Different cut-off values have been adopted in different trials for the definition of “high TMB”^23^, and the possibility that TMB might not have homogeneous predictive power across all malignancies has not been considered^34^. More importantly, although some studies have used whole-exome sequencing (WES) to calculate TMBs^35,36^, this technique cannot be easily applied in the clinical setting due to its high cost, material needs, long turnaround time, and complicated bioinformatics pipelines. In consequence, gene panels targeting around 1 MB (3% of the total exome) are currently being employed to estimate TMB in all types of solid tumors. Two examples are the Foundation One (FO)^37^ and the MSK-Impact^34^ panels, which cover the exomes of 315 and 468 cancer-related genes. Although these panels have demonstrated some predictive power for clinical benefit to ICB therapy^24,28,34,38^, they were designed to detect driver mutations and other biologically relevant genetic alterations, not to calculate TMB. To date, to the best of our knowledge, bioinformatic tools have been rarely applied for the rational design of gene panels to accurately extrapolate TMB, and the results have never been clinically validated.

Here, we present a bioinformatics-based approach for the selection of gene panels and associated models to determine TMBs. First, we studied the distribution of mutant genes and found a high variability across the different human tumors. Consequently, a cancer-specific strategy was employed where relevant genes were selected and used to generate panels by a multiple linear regression algorithm. The TMB predicted by these panels was compared with experimental TMBs, the smaller panels with good accuracy were subjected to rigorous internal and external validation, and those showing the best performance were finally selected. As a result of this analysis, we propose cancer-specific panels for 14 malignancies and we demonstrate that these panels, which range between 0.23 and 1.49 Mb (33 and 231 genes, respectively), can reliably estimate TMBs.

## Results

### Distribution of TMB across cancer types

**Fig. 1:**
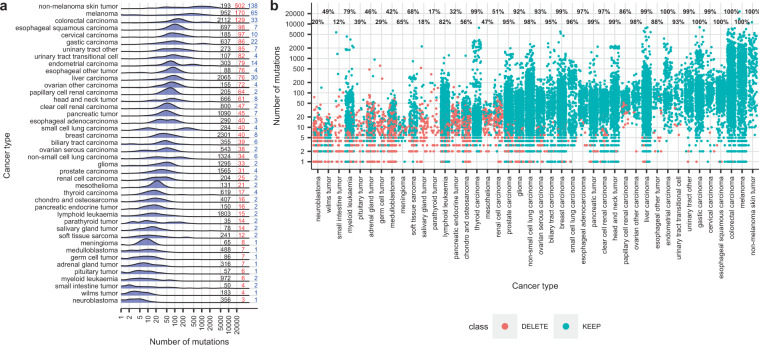
Mutations in the tumor samples of COSMIC v84 (training dataset). **a** Distribution of TMBs across 42 cancer types in the 24,726 samples of the training dataset. The “Y” axis corresponds to the frequency of mutant samples. Numbers on the right of the plot indicate: black, number of samples included in the analysis; red, median TMBs; blue, median of common mutant genes. Cancer types are ordered by median TMBs. **b** Number of mutations in the individual tumor samples of COSMIC v84. Each dot represents a sample. In blue, samples conserved for the development of panels for TMB prediction, in red samples discarded. Numbers on top of each cancer type are the percentage of conserved samples.

The aim of our study was to rationally select panels, with a limited number of genes or exons, which could be used to predict TMB using appropriate mathematical models. To this aim, we first analyzed the distribution of experimental TMBs in our training dataset of 24,726 tumor samples from 42 cancer types with WES or WGS data available in COSMIC_v84 (Fig. 1a). COSMIC is the most extensive mutation database in cancer and, since 2010, integrates the samples from The Cancer Genome Atlas Project (TCGA), the International Cancer Genome Consortium (ICGC), and the Cancer Genome Project (CGP)^39,40^. Remarkable differences were observed in median TMBs of COSMIC_v84 samples, which showed a >100-fold variation, ranging from 3 in autonomic ganglia to 502 in skin carcinoma. In addition, some tumor types presented an apparently unimodal distribution of TMBs, while bimodal distributions were observed in others, such as stomach, endometrium, or lung small cell and non-small cell carcinomas. The distribution of common mutant genes across samples was also analyzed. Although some malignancies such as skin carcinoma, melanoma, large intestine or liver carcinoma showed a median of more than 30 mutant genes in common between individual tumors, in most cancer types this percentage ranged from 0 to 10 (blue numbers in Fig. 1a).

### Development of panels for TMB prediction

**Fig. 2:**
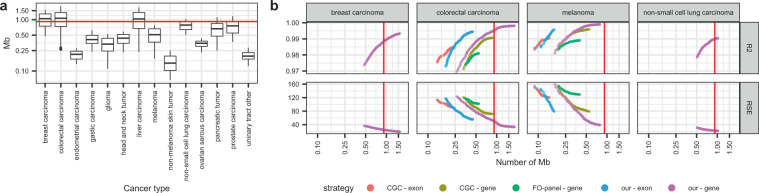
Characteristics of the panels for TMB prediction. **a** Size (in Mb). The red line indicates the size of the FO-panel. The center line in the boxes corresponds to the median, the bounds of the boxes to the first and third quartiles, and the whiskers extend to the largest and lowest values no further than 1.5 times the inter-quartile range. Data beyond the end of the whiskers are called “outlying” points and plotted individually. **b** *R*^2^ and RSE of the panels vs. size (in Mb). Only 4 representative cancer types are shown; breast carcinoma, colorectal carcinoma, melanoma, and non-small cell lung carcinoma (for other tumors, see Supplementary Fig. 4). The red line indicates the size of the FO-panel. Each dot represents a model, colors indicate initial datasets.

In view of the significant variability among different types of tumor, a cancer-specific strategy was used to design panels for TMB prediction (Supplementary Fig. 1). After excluding the largest genes, we selected for each cancer type the genes with mutations in ≥1% of the samples. Samples with mutations in at least one of these “gene-sets” were used for further analysis (Fig. 1b). The “gene-sets” were submitted to a multiple linear regression algorithm in order to generate panels and associated mathematical models. The TMB predicted using these panels and models was compared with experimental TMBs and panels with the minimum number of genes and good accuracy were used to design cancer-specific consensus panels. Consensus panels were then subjected to rigorous internal and external in silico validations and those showing the highest accuracy were finally selected (see the “Methods” section). As a result of this process, we obtained 1446 panels and associated models for 14 cancer types that allow for rigorous prediction of TMB. The 14 cancer types include the most common solid malignancies, such as non-small-cell lung carcinoma (NSCLC), colorectal carcinoma (CRC), melanoma, prostate, and breast carcinoma. The total number of panels per cancer type, as well as the number of Mb and genes that should be sequenced in order to estimate TMBs, are shown in Table 1 and Fig. 2a; while Fig. 2b shows the correlation (R2) with experimental TMBs and the error (expressed as RSE) of the TMBs predicted according to our panels.

**Table 1:** Characteristics of the total (left) and suggested panels (right, bold) for accurate prediction of TMB in 14 cancer types.

Type of tumor	Number of panels	Genes (range)	Mb (range)	Suggested panel Genes	Suggested panel Mb
Breast carcinoma	161	130 (48–210)	1.05 (0.48–1.56)	**199**	**1.49**
Glioma	68	63 (21–101)	0.34 (0.11–0.52)	**98**	**0.52**
Endometrial carcinoma	25	28 (16–40)	0.21 (0.14–0.28)	**33**	**0.23**
Colorectal carcinoma	280	173 (33–323)	1.06 (0.26–1.81)	**185**	**1.10**
Liver carcinoma	250	150 (26–278)	1.30 (0.23–1.74)	**231**	**1.48**
Non-small cell lung carcinoma	107	130 (77–185)	0.79 (0.51–1.02)	**175**	**0.97**
Ovarian serous carcinoma	26	52 (34–67)	0.35 (0.23–0.43)	**61**	**0.40**
Pancreatic tumor	131	19 (17–31)	0.66 (0.25–1.11)	**111**	**0.76**
Prostate carcinoma	108	103 (49–170)	0.76 (0.37–1.17)	**124**	**0.90**
Non-melanoma skin carcinoma	31	24 (9–40)	0.14 (0.07–0.25)	**40**	**0.25**
melanoma	110	70 (15–126)	0.51 (0.17–0.79)	**126**	**0.79**
Gastric carcinoma	68	63 (30–99)	0.41 (0.24–0.62)	**80**	**0.49**
Head and neck tumor	58	67 (36–96)	0.44 (0.23–0.59)	**89**	**0.56**
Urinary tract other	23	30 (19–41)	0.20 (0.13–0.28)	**40**	**0.28**

Bold values indicate the number of genes and size of the only panel for TMB estimation we suggest for each malignancy.

Next, we applied the same strategy to “our exon-sets” and to the genes and exons of the FO panel and the Cancer Gene Census (CGC). The FO and CGC sets allowed us to generate valid consensus panels only for 3 and 8 cancer types, respectively. Models based on “our gene-sets” systematically predicted TMB with more accuracy than models based on the genes or exons of the FO-panel or CGC (*p* < 0.05 in Kruskal–Wallis and *p* > 0.05 in Dunn tests; Supplementary Data 1). Also, models based on genes were systematically superior to models based on exons (Supplementary Figs. 2 and 3).

Due to different reasons, we could not generate panels for TMB prediction in 28 out of the 42 initial cancer types. Some tumors (*n* = 12) had an insufficient number of samples after data curation. In other cases (*n* = 5), the models and panels obtained did not qualify to generate consensus models. Finally, consensus models for some cancer types did not pass the internal (*n* = 5) or external (*n* = 6) validations due to high error (RSE) or low correlation (*R*2) with experimental TMB.

### Overlap between panels

**Fig. 3:**
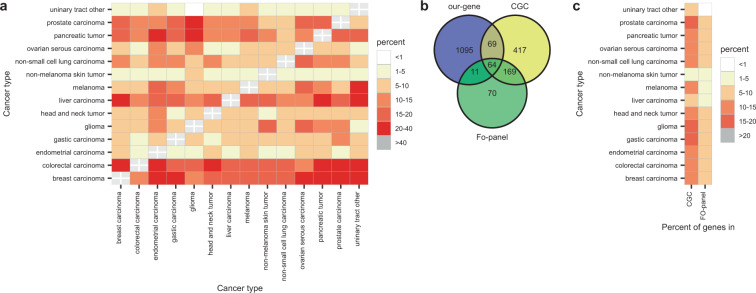
Gene overlap between panels. **a** Percentage of shared genes between the panels for TMB prediction based on “our gene-set”. **b** Euler-Venn diagram of genes in the panels for TMB prediction based on “our set”, CGC genes and genes in the FO-panel. **c** Percentage of shared genes between panels for TMB prediction, CGC and FO-panel, per cancer type.

Next, we analyzed the overlap of the panels for TMB prediction generated from “our gene-sets”. The percentage of genes shared between the panels for different tumors ranged from 0 to 40% and, in some cases such as urinary tract or non-melanoma skin tumor, never reached 10% (Fig. 3a). Then, we compared the panels for TMB prediction with the 314 genes of the FO-panel and the 719 genes of the CGC. Most of the genes in our panels were not contained in the FO-panel or in the CGC (Fig. 3b). When discriminating by cancer type, the overlap was always <20% (Fig. 3c).

### Suggested panels and models and their correlation with response to immunotherapy

In order to facilitate implementation in the clinical setting, we made a final selection of one panel and associated model for each of the 14 cancer types mentioned above. These best panels showed optimal performances in terms of correlation with experimental TMB and low error, having sizes between 0.23 and 1.49 Mb (Table 1 and Supplementary Data 2).

**Fig. 4:**
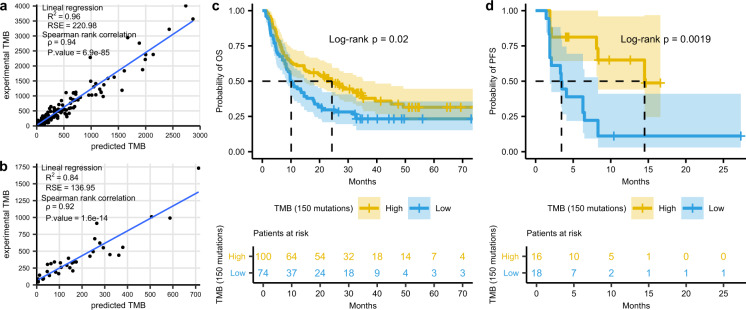
Predicted TMB and response to immunotherapy. **a**, **b** Correlation between experimental TMB and the TMB predicted according to our suggested panels for two reported cohorts of (**a**) melanoma (*n* = 174) and (**b**) NSCLC (*n* = 34) patients treated with ICB therapy (two melanoma samples with >6000 mutations are omitted for clarity). **c** Kaplan–Meier plot of OS in the 174 metastatic melanoma patients, stratified according to predicted TMB (cut-off, 150 mutations). **d** Kaplan–Meier plot of PFS in the 34 advanced NSCLC patients, stratified according to predicted TMB (cut-off, 150 mutations).

To evaluate the association of the TMBs estimated with our suggested panels and outcome to ICB therapy, we used two published cohorts of metastatic melanoma (*n* = 174) and advanced NSCLC (*n* = 34). First, we predicted the TMB for each sample using our suggested panels of 126 genes (0.79 Mb) for melanoma and 175 genes (0.97 Mb) for NSCLC. As expected, predicted TMBs showed an excellent correlation with the published, experimental TMBs (*R*2 = 0.96 and 0.84 for melanoma and lung, respectively; Fig. 4a–b). Regarding the response to ICB therapy, we evaluated three different cut-off values for predicted TMB; 100, 150, and 200 mutations. The 150 threshold showed the best correlation with clinical outcomes (Supplementary Figs. 4–6). Response rate of melanoma patients with >150 predicted mutations was 51%, compared with 18% in those with <150 predicted mutations (*p* < 0.05 in a z-score test) (Supplementary Fig. 7). In addition, patients with high predicted TMB had a median OS of 24.3 months, significantly longer than the 10.1 months of patients with low predicted TMB (HR = 0.66; CI95% = 0.44–0.93; *P* = 0.02) (Fig. 4c). In the case of the NSCLC cohort, the 150 cut-off value also had the best performance (Supplementary Fig. 8), with 56% of patients with high predicted TMB showing partial responses to ICB therapy, compared to 17% in patients with low TMB (*p* < 0.05 in a z-score test) (Supplementary Fig. 7). Only progression-free survival (PFS) but not OS data was available for this cohort. Despite the relatively low number of patients, the median PFS of those patients with >150 predicted mutations was significantly longer, 14.5 months vs. 3.5 months for patients with low predicted TMB (HR = 0.27; CI95% = 0.10–0.57; *P* = 0.002) (Fig. 4d).

The performance of predicted TMB to evaluate clinical benefit was also analyzed using ROC curves (Supplementary Figs. 9–10). The areas under the curve (AUC) (0.67 for the melanoma and 0.70 for the NSCLC cohort) were similar to those obtained when using experimental TMBs (0.67 and 0.76, respectively).

## Discussion

TMBs should ideally be determined by WGS or WES but the widespread implementation of these techniques in the clinical setting is difficult due to long turnaround time, complexity, cost, and tissue consumption. In consequence, NGS panels^34,37^ ranging from 1.1 to 3 Mb are increasingly used to estimate TMB in tumor samples, and two of them have recently received FDA approval after demonstrating some predictive power in several malignancies^23^. However, the majority of NGS panels in clinical use have not been designed to extrapolate TMB. Instead, they target genes carrying driver mutations or other clinically relevant alterations, which are not necessarily representative of the total number of mutations in the entire exome. Furthermore, the same NGS panels are used in all solid tumors, ignoring the fact that mutant genes are not the same and do not present the same frequency of mutation across different malignancies (Fig. 1). Finally, TMB estimates based on NGS panels are subjected to biases that can derive in the misclassification of up to 19–21% of samples when using large panels (1.1–1.4 Mb), or up to 33–36% for smaller panels^41^. In consequence, the development and validation of gene panels for accurate estimation of TMB is of particular interest. However, to the best of our knowledge; rational, bioinformatics-based strategies have rarely been attempted to achieve this aim.

Here, we present a data-driven strategy that allowed us to develop cancer-specific panels and associated mathematical models for accurate prediction of TMB in 14 types of tumors; including the most common malignancies such as NSCLC, CRC, breast carcinoma, melanoma or prostate carcinoma. These panels, which were submitted to rigorous internal and external in silico validations, range from 0.23 to 1.49 Mb, a size comparable with the panels currently used in the clinical setting (0.9 Mb for FO and 1.22 Mb MSK-Impact). A recent report^42^ concluded that 1.5–3 Mb panels are better suited to calculate TMB, whereas smaller panels can yield considerably imprecise estimates. However, we have demonstrated that the <1.5 Mb panels selected using our bioinformatics-based strategy can accurately predict TMB (Fig. 2a).

Several studies have reported the development of methods for TMB calculation based on commercial NGS panels^43,44^. However, to the best of our knowledge, there is only one study where “in silico” tools were used to select genes for TMB prediction^45^. As a result, the authors proposed a 525 gene pan-cancer panel, which is significantly larger than the 33 to 231 gene cancer-specific panels we suggest (Supplementary Data 2). In some cancer types, this 525 gene panel showed an average correlation with experimental TMB (*R*^2^) comparable to our panels. However, the variability in the error of the samples (RSE), which can lead to sample misclassification, was not reported by the authors. In addition, the 525 panel was not validated with clinical data to determine its performance in terms of prediction of clinical outcome to ICB therapies. Finally, the common genes among our suggested tumor-specific panels range from 1 to 40%, indicating that a single selection of genes is not optimal to estimate the TMB due to the heterogeneity of mutant genes across the spectrum of human malignancies.

During our analysis we also found that, although commercial NGS panels can offer acceptable estimations of TMB in certain malignancies, they are not optimal for this purpose. The cancer-specific panels we suggest outperform the FO panel and the CGC gene set for TMB estimation (Fig. 2, Supplementary Fig. 2). For some cancer types, only our set of genes generated acceptable models for TMB prediction while for other malignancies, although sometimes FO or CGC-based estimates were acceptable, our panels showed a significantly better *R*^2^ and lower RSE. We also found most of the best genes for TMB calculation are not included in the FO panel or CGC set, being the overlap <20% (Fig. 3b–c). These findings further support the idea that panels composed of oncogenes and tumor suppressor genes systematically introduce biases and have a higher variance than those based on a random selection of genes^42^.

In our study, TMB was considered as the total number of exonic somatic mutations; a definition aligned with the scientific literature and published clinical trials. Although the analysis of variants in non-coding regions might be of interest, we excluded them for several reasons. The non-coding segments of the genome are much longer than the exons, the number of possible mutations is enormous and a vast majority of them are probably silent, unlikely to generate neoantigens. Also, the number of samples with whole-genome sequencing (WGS) and clinical data available is relatively small, probably insufficient to obtain statistically significant results. Therefore, testing if a “WGS TMB” can be superior to the commonly used “WES TMB” would require a comprehensive study, where WGS should be performed in a significant number of patients receiving ICB therapy with response data available.

Clinical validation of our results in a prospective study would add considerable value to our work. However, in the 14 cancer types where we could select panels and models, our predicted TMB highly correlated with the experimental TMB. Also, the TMBs calculated using our suggested panels closely correlated with the TMB experimentally obtained by WES in two small cohorts of melanoma and lung cancer patients with publicly available data (Fig. 4). In addition, when patients were classified according to our estimates, response rates were 51–56% for the high TMB vs. 17–18% for the low TMB group. Both values are in excellent agreement with the 46–59% vs. 12–23% reported in several clinical trials of ICB therapies in lung and melanoma tumors where TMB was determined by WES^24,46–48^ and outperform the 20–45% vs. 4–25% response rates obtained when the FO or MSK-Impact panels were employed^23,28,49^. Regarding survival, patients with high predicted TMB according to our models showed 14.5 months PFS in the lung and 24.3 months OS in the melanoma cohorts; compared to 3.5 and 10.1 months respectively for patients with low TMB. Again, these values are in the range of the figures reported in the literature for patients stratified according to WES data; 7.1–17.1 months for high TMB lung and 24–25 months for high TMB melanoma vs. 1.4–3.7 and 9–10 months, respectively, for patients with low TMB^24,46–48^.

In summary, we have developed a new bioinformatics-based method to select NGS panels and associated mathematical models for accurate prediction of TMB, which we have validated using clinical data. The panels we suggest are tumor-specific, can be easily implemented in the experimental setting and outperform the driver-oriented NGS panels currently used to estimate TMB in tumor samples. In addition, the TMB predicted by our panels correlates with clinical outcome to ICIs. Our work can facilitate a more accurate prediction of TMB which, in turn, can improve the discrimination between patients responding and not responding to ICB therapy.

## Methods

### Training dataset

We downloaded the WGS and WES datasets of the Cosmic version 84 (Cosmic_v84)^39^ for human grch38 assembly, which contained 25,533 tumor samples classified into cancer types according to the primary site, primary histology, and histology subtype. Then, we excluded (i) benign tumors, (ii) samples with mutations without annotation of genomic coordinates, (iii) samples with non somatic mutations (labeled as “Variant of unknown origin” or “Not specified”), (iv) samples with mutations exclusively in non coding regions. Finally, cancer types with <10 samples were also excluded. Our training dataset contained 24,726 samples of 42 cancer types. For detailed information of samples and filtering see Supplementary Data 3.

### External validation dataset

The external validation dataset was based on the new WGS and WES samples added to COSMIC_v90 (*n* = 3144), together with samples with WGS and WES data retrieved from 133 articles publicly available in the literature (*n* = 4773) (Supplementary Data 4). All samples were mapped to the grch38 genomic coordinate and filtered according to the procedure described for the training dataset (Supplementary Data 1). Overall, the external validation dataset contained 7917 samples from 40 cancer types. Parathyroid and pituitary cancers were not represented.

### Study population in the clinical validation set

We used 174 metastatic melanoma^35,36^ and 35 NSCLC^24^ patients (Supplementary Data 3) with anonymized WES data and clinical outcome to ICB therapy publicly available. All patients had been enrolled in clinical trials outside of our institution. The patients had signed informed consent under institutional review board (IRB) approved protocols. In addition, the results of the genetic testing and outcome to ICIs had been previously published by the corresponding investigators. No additional human clinical data was used. For these reasons, the IRB of the Quirón Dexeus University Hospital granted a waiver for this study.

### Outline of the development of panels and models for TMB prediction

TMB was defined as the total number of exonic mutations in a given sample. Consequently, only mutations in exons were considered. Our aim was to select cancer-specific panels, with a limited number of genes or exons, which could be used to predict TMB using appropriate mathematical models (Supplementary Fig. 1). First, we excluded from our training database the largest genes (11 genes >18928 bp; 0.05% of the genome), some of which had been also removed or penalized by length in the previous studies^50^. Next, for each cancer type, we selected all genes harboring mutations in at least 1% of the samples and obtained what we called “our gene-sets” and “our exon-sets”. These sets were the input for developing gene and exon models, respectively. Regarding samples, those with mutations in at least one of “our genes” were selected. We found that some cancer types had <80% of samples with mutations in at least one of the genes of “our” sets (Fig. 1b and Supplementary Fig. 2). Therefore, they were excluded from further analysis. For the rest of the cancer types, we generated panels to predict TMB using multiple linear regression algorithms that assigned appropriate coefficients to each gene. Predicted TMB accuracy was assessed by the coefficients of correlation (*R*2) and relative standard error (RSE) when compared with experimental TMB. Panels with the minimum number of genes, *R*2 > 0.6 and RSE lower than the median TMB by cancer type were subsequently used to generate cancer-specific consensus panels and to develop associated models. Then, consensus panels and models were subjected to internal and external validation and those showing the highest *R*2 and lowest RSE were finally selected (Supplementary Data 5). For comparison purposes, we applied the same procedure to the genes of the FO-panel and the CGC^51^. A detailed description of the development of cancer-specific panels and models for TMB prediction is presented in the sections below.

### Selection of genes and samples

Within each cancer type, genes were ordered according to the number of mutant samples and the top 10 genes were selected. A total of 157 different genes appeared in this analysis, including, *TP53*, *TTN*, *MUC16*, *MUC4*, *KRAS*, *PIK3CA*, *SYNE1*, *APC* or *OBSCN*, (Supplementary Fig. 11a). Some of them were long genes codifying for large proteins, such as *TTN* and *MUC16*, which have coding regions of 109 and 44 Kb, respectively. In consequence, similarly to the previous studies^50^, we penalized genes by length (see below). After the penalization, 57 genes no longer appeared among the top 10 genes most frequently mutated by cancer type, including *TTN* or *MUC16*; while and 100 genes remained, such as *TP53*, *KRAS*, *PIK3CA*, *BRAF*, or *APC* (Supplementary Fig. 11b).

The human genome assembly Grch38 comprises 20,291 coding genes, which were obtained using the “gtf data” from the Ensembl version grch38.91. For each gene, the number of coding bases was calculated; the median length was 1341 bp; Q1 and Q3 were 828 and 2139 bp, respectively. Among the 20,291 coding genes of the human genome, 499 (2.46%) showed a disproportionate large size of the coding region (Supplementary Fig. 12), higher than three times the interquartile distance (6072 bp), and were considered extreme outliers. To avoid the bias due to the gene length, the number of mutant samples per gene (G) and cancer type (C) was corrected as shown in Eq. 1. For example, the FLG gene has 12,186 coding bp and 379 mutant samples among large intestine tumors. After applying the equation, they counted as 188 mutant samples [379/(12,186/6072)]. The equation did not penalize genes with <6072 coding bp.

1 $${\mathrm{samples}}\,\mathrm{penalized}\,\;\left( {G,C} \right) = \frac{{\mathrm{samples}\,\mathrm{in}\,C}}{{\max \left( {1,\frac{{\mathrm{coding}\,\mathrm{base}\,\mathrm{pairs}\,\mathrm{of}\,G}}{{6072}}} \right)}}$$

In order to develop the cancer-specific panels for TMB prediction, we selected the genes with mutations in >5 samples representing >1% of the total number of samples in a particular cancer type; generating what we called “our gene-sets”. Regarding samples, those with mutations in at least one of “our genes” were selected. Some cancer types had <80% of samples with mutations ≥1 of the genes in “our set” and were excluded from further analysis (Fig. 1b). The final number of samples and cancer types selected for further analysis is presented in Supplementary Data 3.

Using a similar strategy, we generated “our exon-sets”. First, to avoid the bias due to the gene length, the number of mutant samples per gene (G), exon (E) and cancer type (C) was corrected according to Eq. 2. Then, we selected the exons with mutations in >2 samples representing >1% of the total number of samples in a particular cancer type; generating “our exon-sets”. Finally, only samples with mutations in at least one of “our exons” were selected (Supplementary Fig. 13 and Supplementary Data 3)

2 $${\mathrm{samples}}\,\mathrm{penalized}\,2\;\left( {E,G,C} \right) = \frac{{\mathrm{samples}\,\mathrm{penalized}\left( {G,C} \right)}}{{\left( {\frac{{\mathrm{coding}\,\mathrm{base}\,\mathrm{pairs}\,\mathrm{of}\,G}}{{\mathrm{coding}\,\mathrm{base}\,\mathrm{pairs}\,\mathrm{of}\,E}}} \right)}}$$

For comparison purposes, we applied the same strategies to the genes and exons of the Cancer Gene Census (CGC)^51^ and the Foundation One panel (FO-panel)^37^. The CGC was downloaded from the Cosmic_v84 version and contained 719 genes. Six of them (*IGH*, *IGK*, *IGL*, *TRA*, *TRB*, and *TRD*) were “macrogenes” and we replaced them with all possible Ensembl gene names within the same genomic coordinates, ending with a dataset of 1080 Ensembl genes. Regarding the 315 genes of the FO-panel, one of them had no coding sequence and was excluded from the analysis. Using the same strategies discussed above, we selected the samples for further analysis (Supplementary Fig. 13, Supplementary Data 3, Supplementary Table 1).

### Generation of initial panels and associated linear models for TMB prediction

A forward step algorithm was used for generating the first set of panels and associated linear regression models for TMB prediction. The accuracy of the panels and associated models was measured with four evaluators. Three of them were metrics; namely R2, adjusted R2, and RSE; the fourth was the sum of the samples selected for the development of the model. The R2 or coefficient of determination was defined as the proportion of the variance in the TMB (dependent variable) that could be predicted from the independent variables (genes/exons in the models). The R2 was adjusted by the number of variables in the models to obtain the adjusted R2. Finally, RSE was defined as the square root of the estimated variance of the random error.

In each step of the iteration, we used the four evaluators to select the 100 best panels and associated models. Then, we deleted the so-called “non-feasible” panels, defined as those having ≥1 gen/exon not statistically relevant. The remaining panels and models were subsequently employed as seeds for the next step. The forward step algorithm ended when it could not add more genes/exons to the panel. All final panels and associated models generated in each step were considered for further analysis; the total number was 731,958 for 30 cancer types. Then, we selected those models with R2 > 0.6 and RSE < median TMB by cancer type; ending up with 354,644 panels.

### Generation of consensus panels and associated models

Due to a large number of panels and associated models obtained as a result of our analysis, we decided to generate cancer-specific consensus panels. Some genes/exons, which only appeared in a few panels, were eliminated to select a core of genes per cancer type according to the following strategy. First, the genes/exons present in the initial panels were ordered by the number of occurrences. Then, they were added in a forward step manner to obtain a series of consensus panels. Those with ≥1 gene/exon with no statistical significance were deleted, leaving a total of 7,242 consensus models in 25 cancer types. These consensus models were submitted to 1000 repetitions of bootstrap internal validation and then to an external validation using samples obtained from a newer version of COSMIC and the literature (see External Validation Dataset, in the “Methods” section). The rules used to select the acceptable consensus panels and the final numbers of consensus panels after each step of the validation are shown in Supplementary Data 3 and Supplementary Table 1. A total of 2301 panels and associated models for 14 cancer types passed the internal and external validation process. Panels developed from “our gene-set” showed the best results in terms of quality parameters (R2 and RSE in the internal and external validations), as demonstrated with the Kruskal–Wallis and posthoc Dunn statistical tests (Supplementary Data 1).

To facilitate the implementation in the clinical setting, we made a final selection of the best panels and associated models for each of the 14 cancer types. We also selected 25 panels with almost optimal performances of different sizes (in Mb). The genes contained in the 14 + 25 panels and their associated models are presented in Supplementary Data 2.

### Clinical validation. Correlation between response to immunotherapy and predicted TMB

First, we evaluated the correlation between experimental and predicted TMB by Spearman’s rank correlation and linear regression in the cohorts of patients mentioned above. Then, the association of predicted TMBs with OS and PFS to ICB therapy was analyzed at cut-off values of 100, 150, and 200 mutations. The correlation between predicted TMB and type of clinical response was also evaluated. SPSS and GraphPad Prism v6.0 were used to generate Kaplan–Meier plots and perform log-rank tests in order to compare OS and PFS data; while ROC curves were made with plotROC^52^ and Venn diagrams with VennDiagram^53^.

### Reporting summary

Further information on research design is available in the Nature Research Reporting Summary linked to this article.

## Supplementary information

Supplementary Information

Supplementary Data 1

Supplementary Data 2

Supplementary Data 3

Supplementary Data 4

Supplementary Data 5

Reporting Summary Checklist

## Data Availability

The data generated and analysed during this study are described in the following data record: 10.6084/m9.figshare.14074451^54^. The datasets and code are available as part of this data record, and also via https://gitlab.com/bioinformatics-fil/predict_tmb. A list of the files underlying the figures, tables and supplementary tables of the related manuscript is available as part of the data record in the file ‘Martinez-Perez_et_al_underlying_data_list.xlsx’. For details about the software used, see the methods of the related publication.

## Code availability

The codes and scripts generated and/or analyzed during the current study are also available at https://gitlab.com/bioinformatics-fil/predict_tmb. All the programming and related statistics were performed with R. The following R libraries were used; Genomic Features^55^, biomaRt1^56^, GenomicRanges, and rtracklayer^57^. Translation databases hg18ToHg38.over.chain and hg19ToHg38.over.chain from UCSC web page (http://genome.ucsc.edu/)^58^ were employed for the filtering datasets process. All graphics were made with ggplot2^59^ Library caret was used for internal and external validation. Wilcoxon test was employed to compare gene and exon models; the Kruskal–Wallis test to compare models obtained using different strategies (our-gene, our-exon, CGC-gene, CGC-exon, FO-panel-gene, and FO-panel-exon) and Dunn Test to identify differences between strategies when the Kruskal–Wallis test was significant.
