# Supplementary material for: Panels and models for accurate prediction of tumor mutation burden in tumor samples
Source: NPJ Precis Oncol. 2021 Apr 13;5:31. doi: 10.1038/s41698-021-00169-0 (PMC8044185; doi:10.1038/s41698-021-00169-0)
Supplement: Supplementary file 1 — Supplementary Information [file 41698_2021_169_MOESM1_ESM.pdf]

Supplementary Material

Panels and models for accurate prediction of tumor mutation burden in tumor samples

Elizabeth Martínez-Pérez, Miguel Angel Molina-Vila and Cristina Marino-Buslje.

SUPPLEMENTARY DATA

Supplementary Data 1. Accuracy of panels and models for TMB prediction

Supplementary Data 2. Suggested panels for TMB prediction in 14 cancer types, classified by Mb to be sequenced

Supplementary Data 3. Filters applied to the training and validation datasets

Supplementary Data 4. External validation dataset

Supplementary Data 5. Filters applied to the consensus panels and models

SUPPLEMENTARY TABLES

Supplementary Table 1. Total number of samples used in the training dataset and types of tumor where >80% samples were included in the dataset**.**

	**Genes**		**Exons**	
**Selection**	**Total samples**	**Types of tumor with > 80% samples**	**Total samples**	**Types of tumor with > 80% samples**
**“our set”**	22166	26	21035	23
**CGC**	22153	30	22153	30
**FO-panel**	20495	23	20495	23

Supplementary Table 2. Number of consensus panels after filtering

**Conditions to be accepted**		**Consensus panels**	**Cancer type**
		7,242	25
i)	R^2 model >= 0.6	6,988	25
ii)	RSE model =< median TMB in each cancer type	6,459	25
iii)	R^2 internal validation >= 0.6 &<br>R^2 internal validation >= R^2 model - 0.1	5,556	22
iv)	RSE internal validation =< median TMB &<br>RSE internal validation =< 1.25*RSE model	2,978	20
v)	R^2 external validation >= 0.6	2,301	14

SUPPLEMENTARY FIGURES

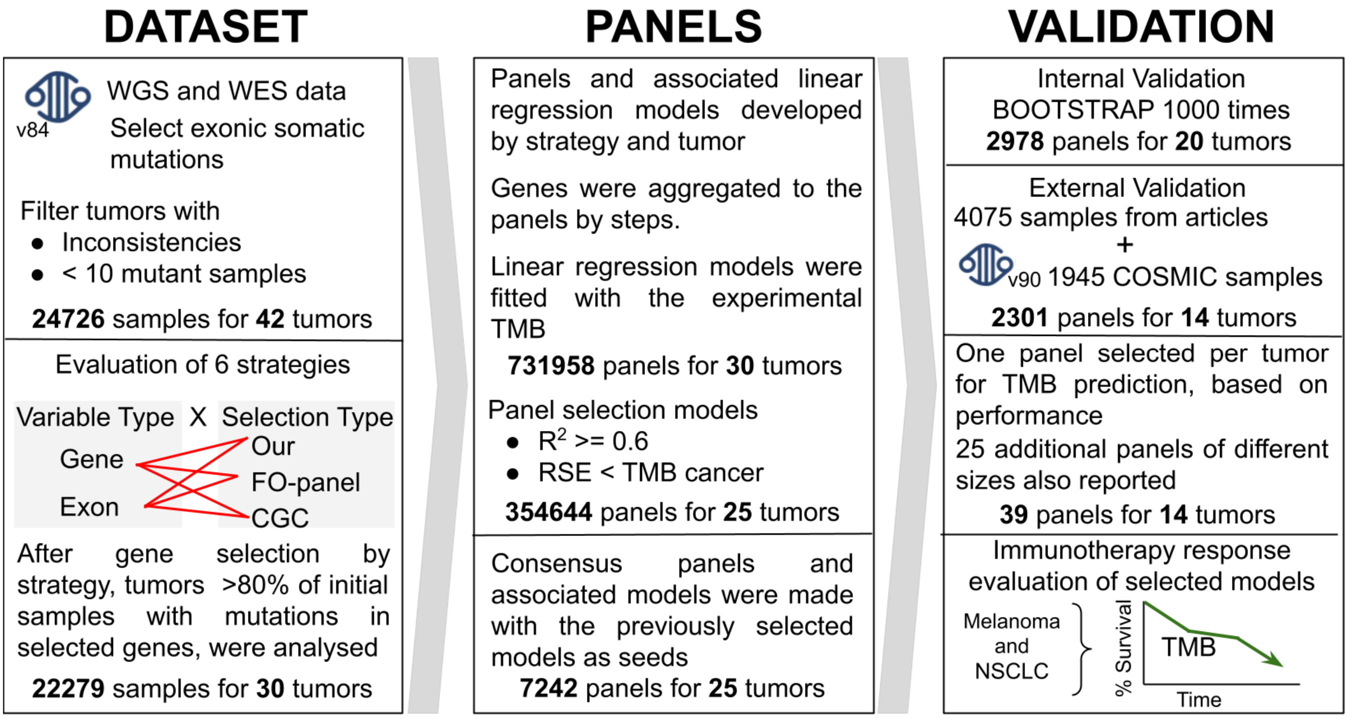

Supplementary Figure 1. Workflow describing the bioinformatics-based approach used to select cancer-specific panels for TMB prediction

a

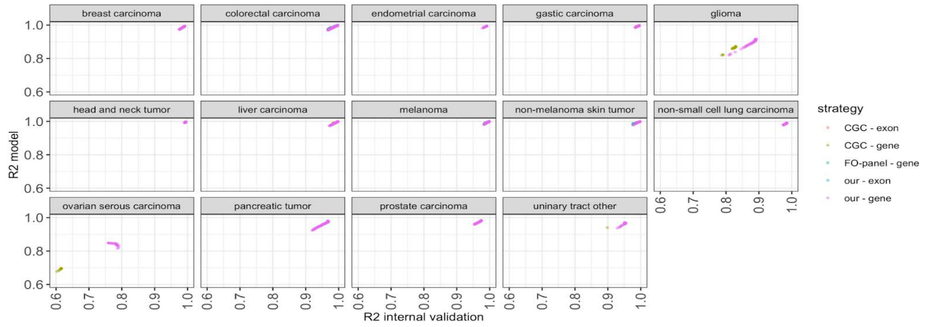

b

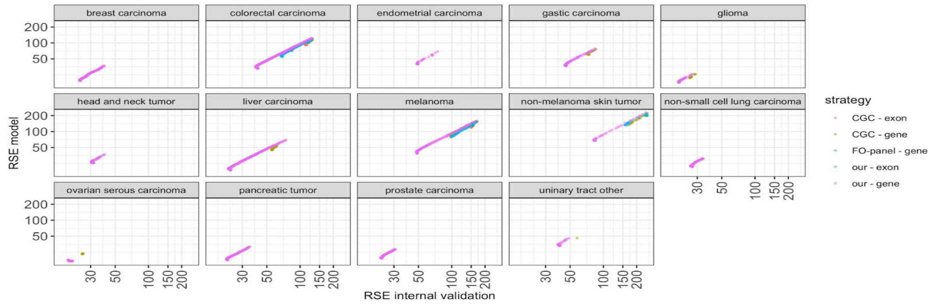

c

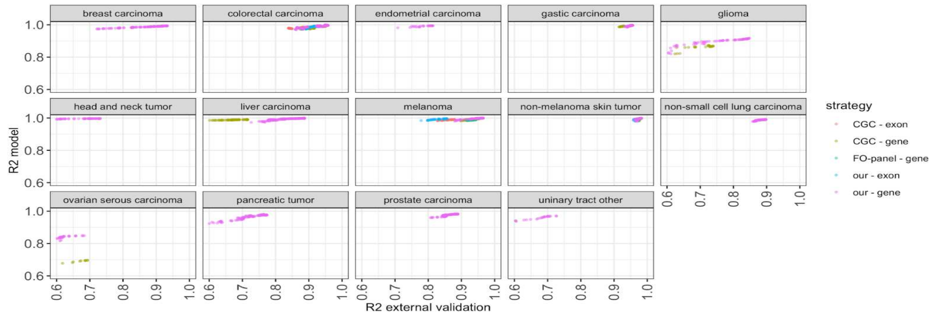

d

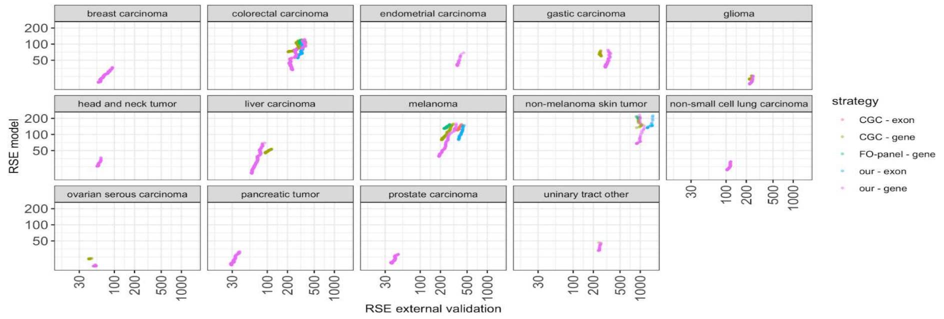

Supplementary Figure 2. Accuracy of the panels and associated models for TMB prediction. a: R^2, internal validation; **b**: RSE, internal validation; **c:** R2, external validation; **d:** RSE, external validation. Each dot represents a model, colors indicate initial datasets.

a

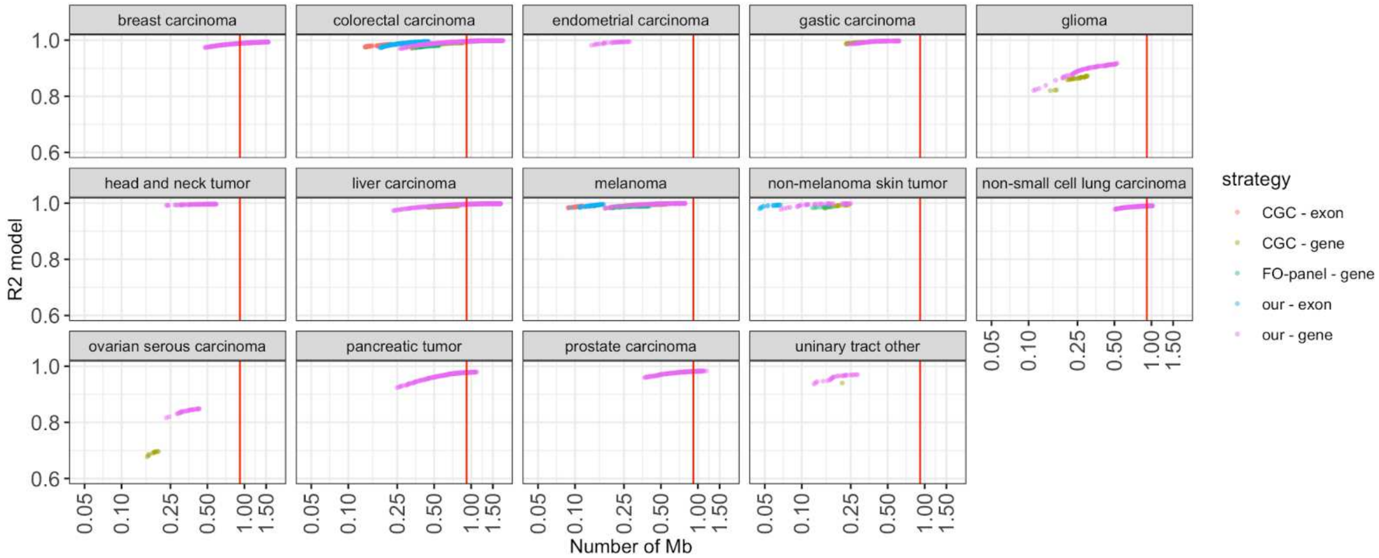

b

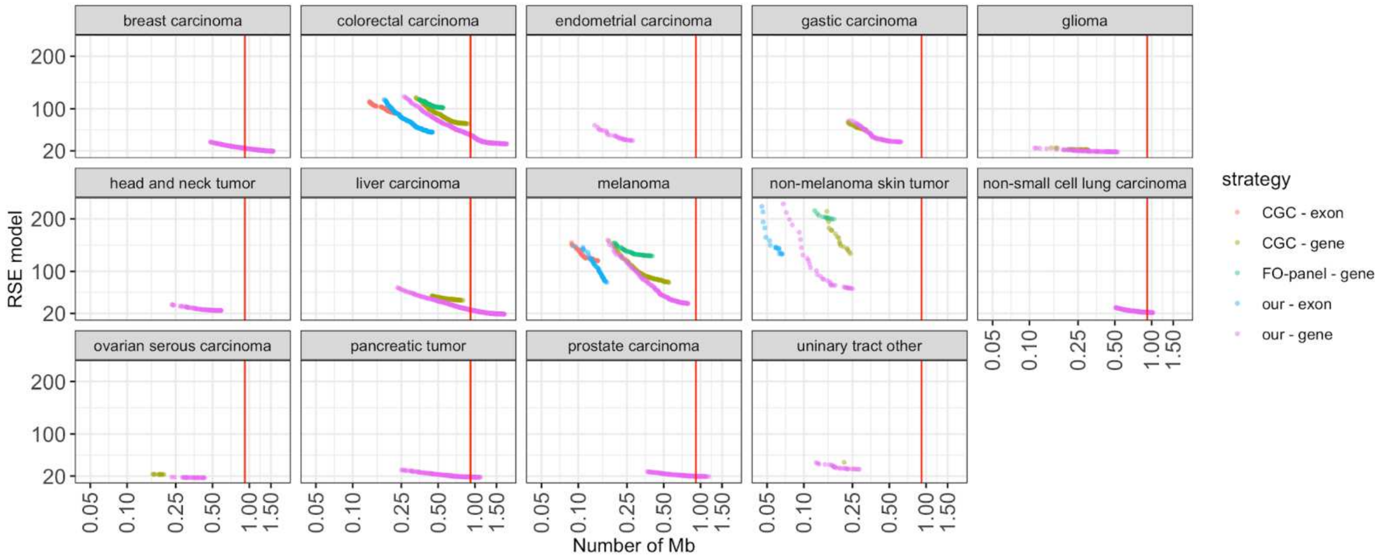

Supplementary Figure 3. Characteristics of the panels for TMB prediction. **a:** R2 and **b:** RSE of the panels vs. length (in Mb). Each dot represents a model, colors indicate initial datasets.

a

Model_119 **Model_80** **Model_42**

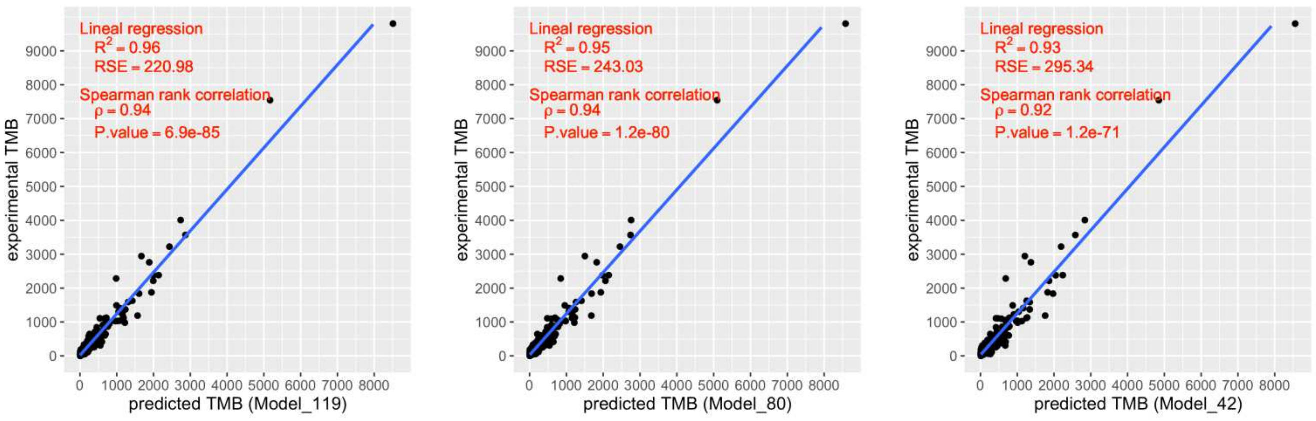

b

Model_115 **Model_73** **Model_37**

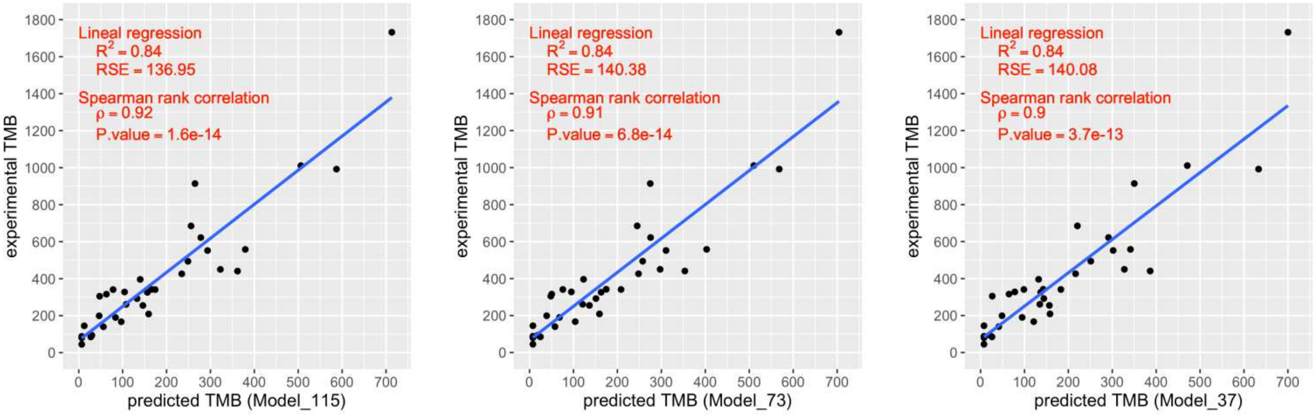

Supplementary Figure 4. Correlation between experimental TMB and the TMB predicted with 3 suggested panels and associated models in two cohorts of melanoma (**a**) and lung-non small cell patients (**b**).

<table>
<tr><th></th><th>100</th><th>150</th><th>200</th></tr>
<tr><td>experimental TMB</td>
<td>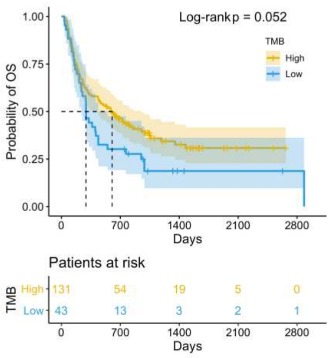
</td>
<td>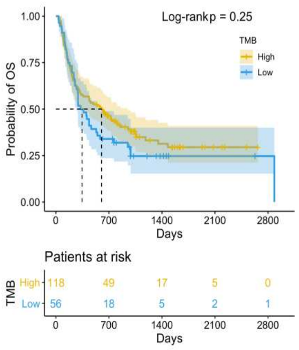
</td>
<td>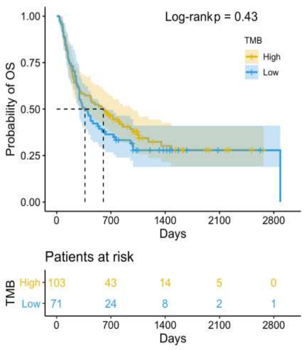
</td></tr>
<tr><td>TMB predicted by model_119 (best model)</td>
<td>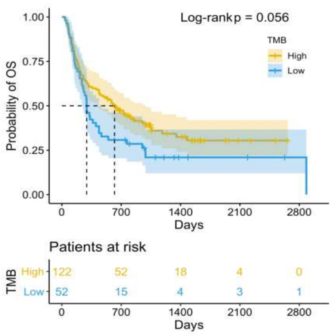
</td>
<td>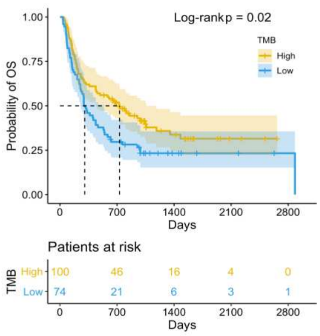
</td>
<td>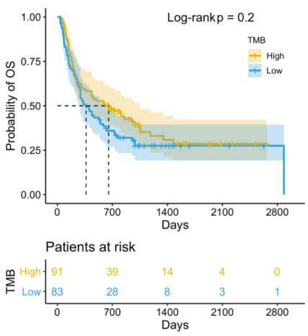
</td></tr>
<tr><td>TMB predicted by model_80</td>
<td>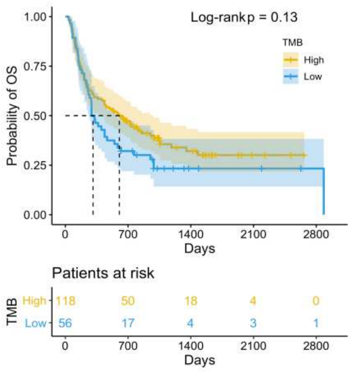
</td>
<td>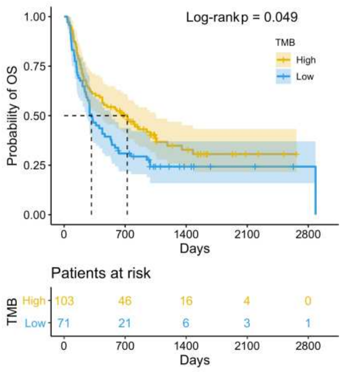
</td>
<td>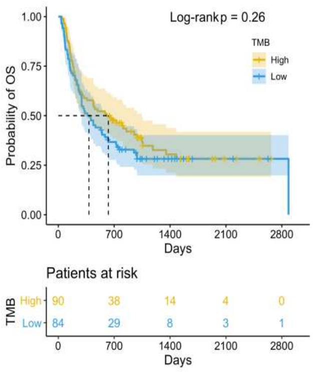
</td></tr>
<tr><td>TMB predicted by model_42</td>
<td>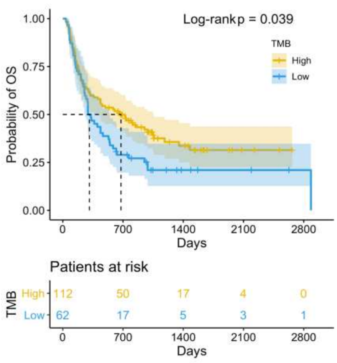
</td>
<td>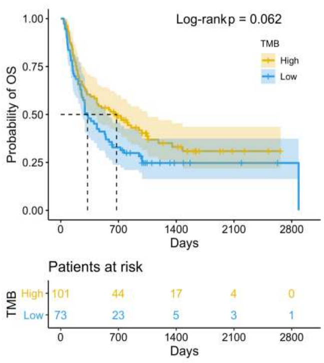
</td>
<td>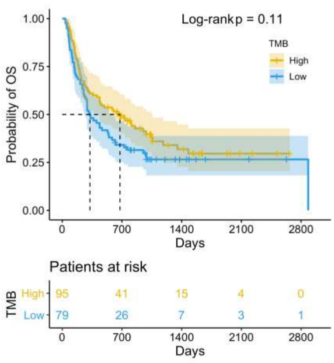
</td></tr>
</table>

Supplementary Figure 5. Kaplan-Meier plot of OS in a cohort of 174 metastatic melanoma patients, treated with ICB therapy, stratified according to predicted TMB with 100, 150, and 200 cut-off values).

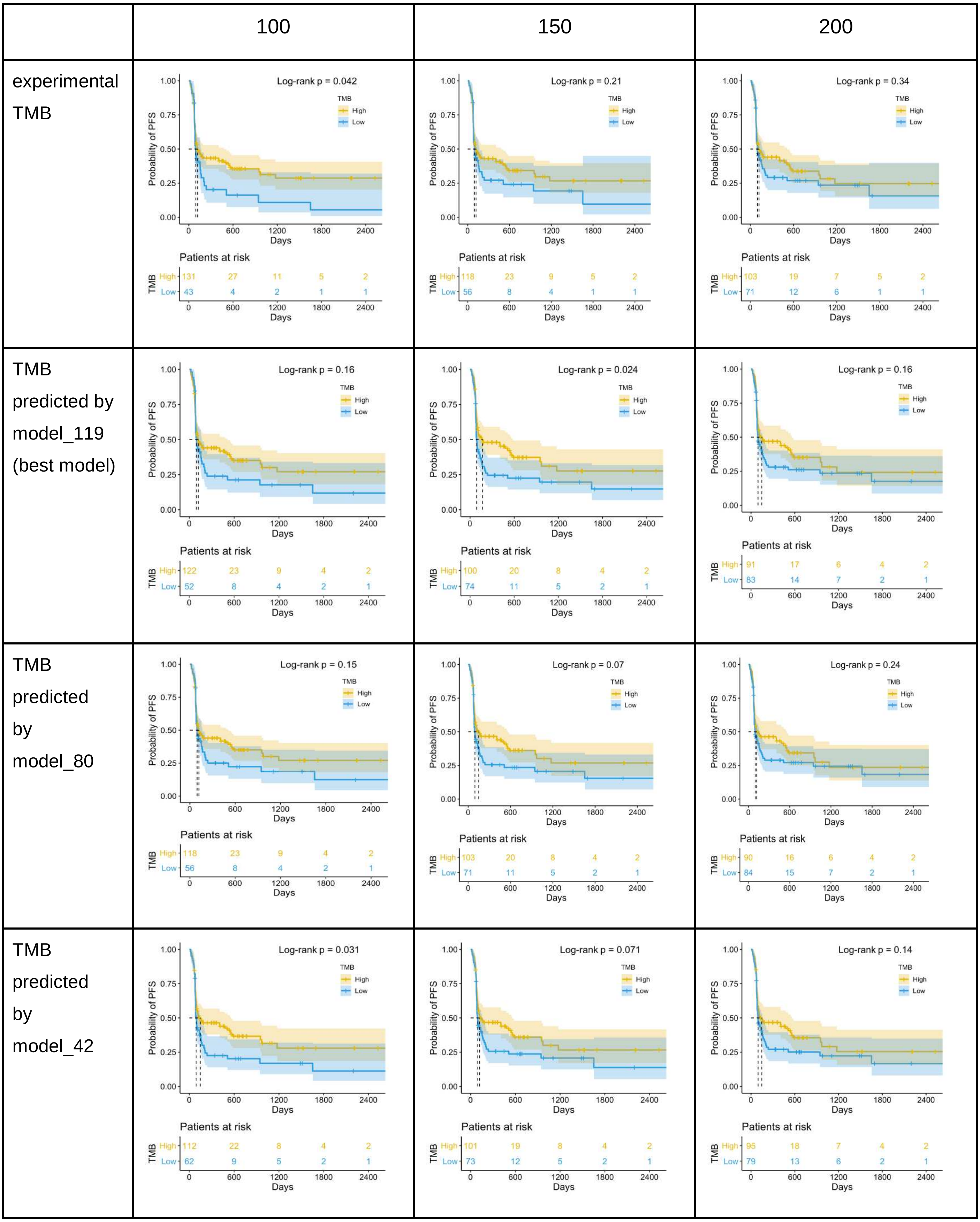

Supplementary Figure 6: Kaplan-Meier plot of PFS in 174 metastatic melanoma patients, treated with ICB therapy, stratified according to predicted TMB (100, 150, and 200 cut-off).

a **b**

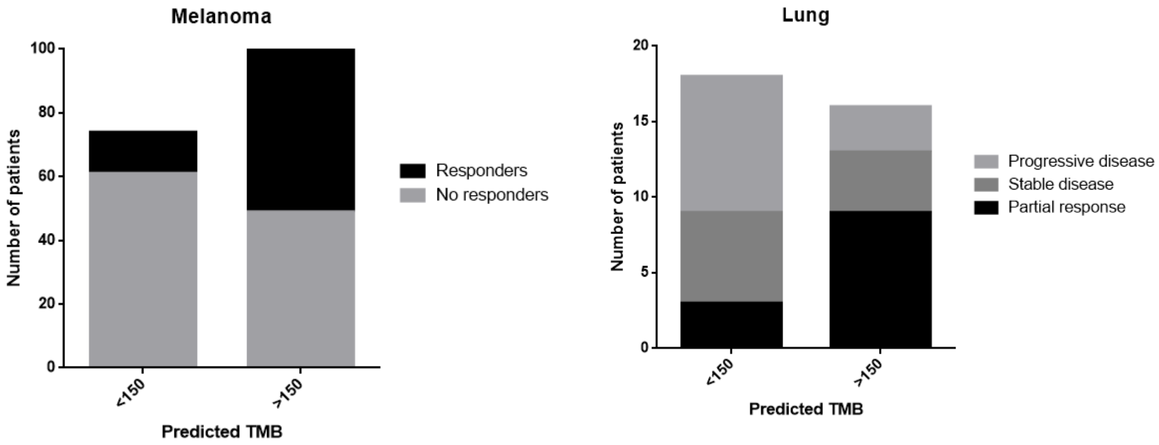

Supplementary Figure 7: Response to ICB therapy to 174 metastatic melanoma (**a**) and NSCLC (**b**) patients, stratified according to predicted TMB (cut-off, 150 mut/Mb)

<table>
<tr><th></th><th>100</th><th>150</th><th>200</th></tr>
<tr><td>experimental TMB</td>
<td>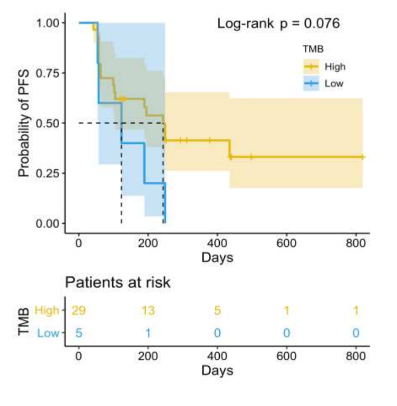
</td>
<td>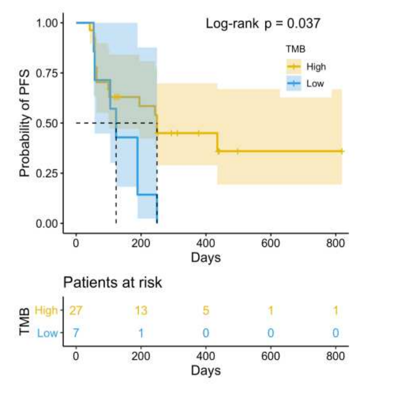
</td>
<td>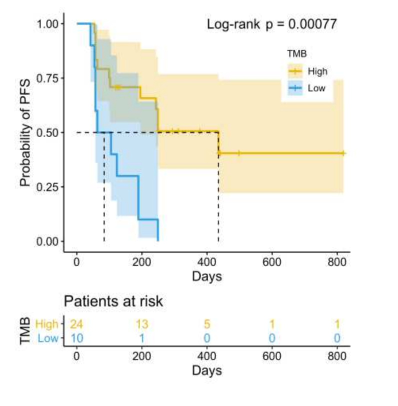
</td></tr>
<tr><td>TMB predicted by model_115 (best model)</td>
<td>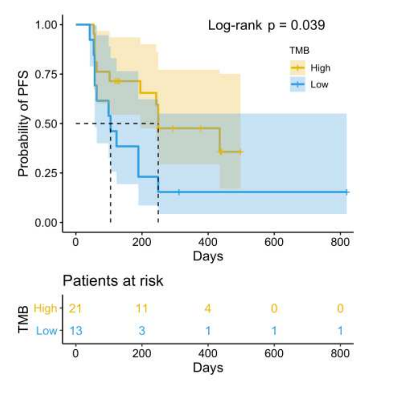
</td>
<td>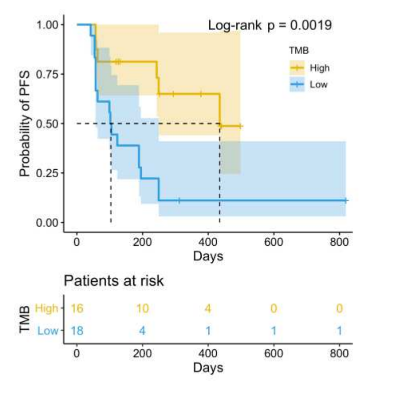
</td>
<td>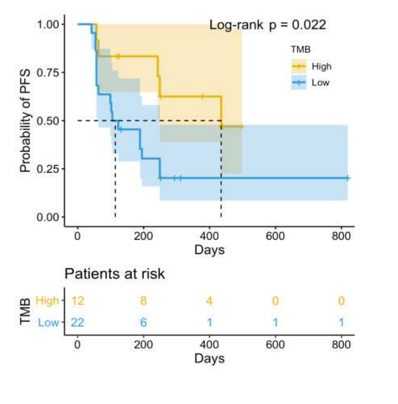
</td></tr>
<tr><td>TMB predicted by model_73</td>
<td>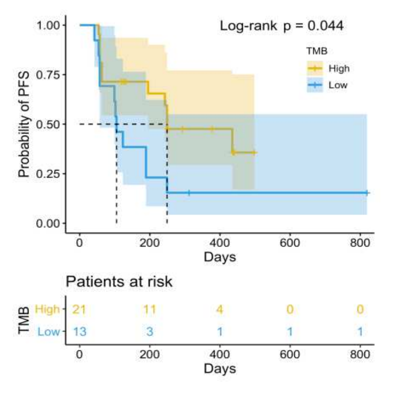
</td>
<td>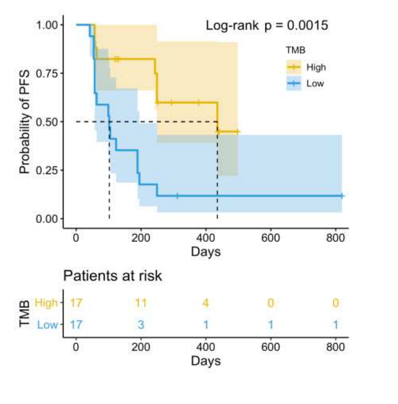
</td>
<td>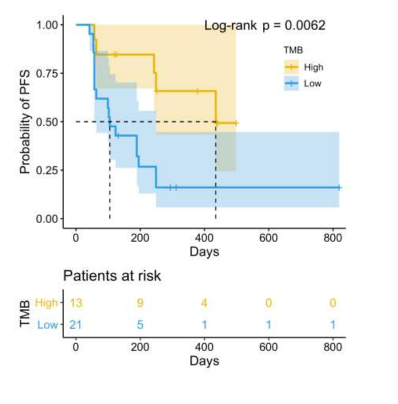
</td></tr>
<tr><td>TMB predicted by model_37</td>
<td>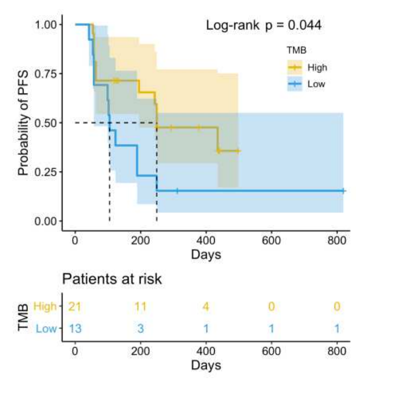
</td>
<td>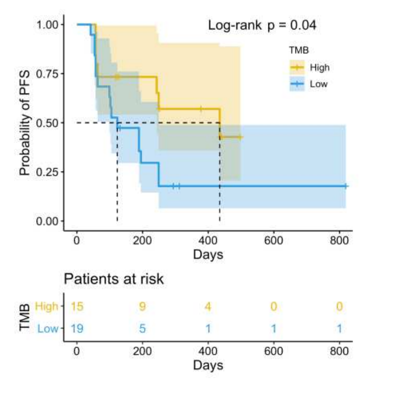
</td>
<td>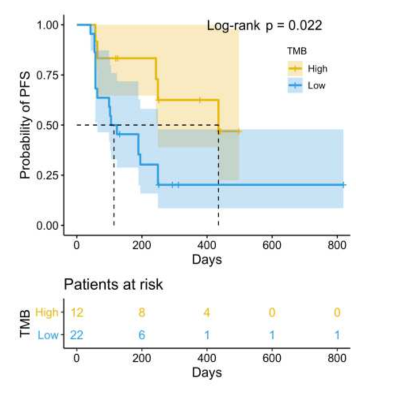
</td></tr>
</table>

Supplementary Figure 8. Kaplan-Meier plot of PFS in 34 metastatic NSCLC patients, treated with ICB therapy, stratified according to predicted TMB (100, 150, and 200 cut-off).

experimental TMB	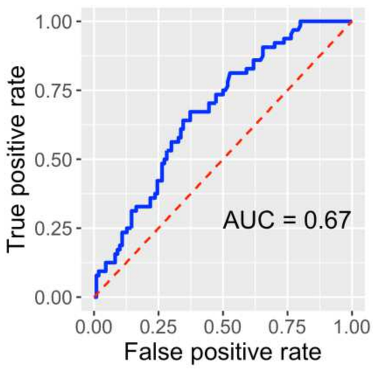
TMB predicted by model_119 (best model)	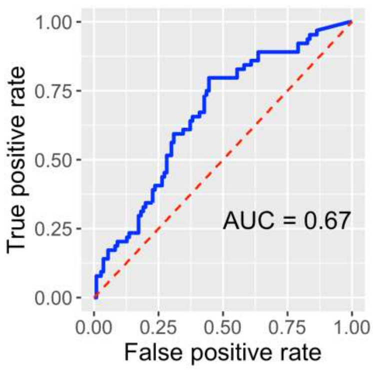
TMB predicted by model_80	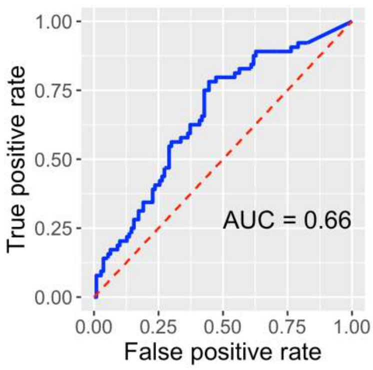
TMB predicted by model_42	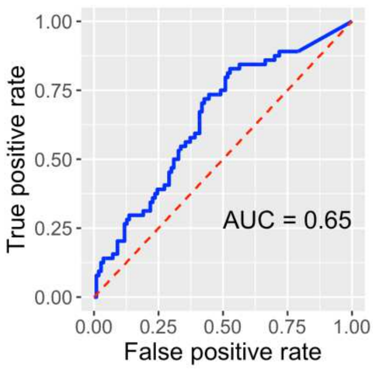

Supplementary Figure 9: ROC curve of predicted TMB and type of response to ICB treatment in a cohort of 174 metastatic melanoma patients

<table>
<tr><td>experimental TMB</td><td>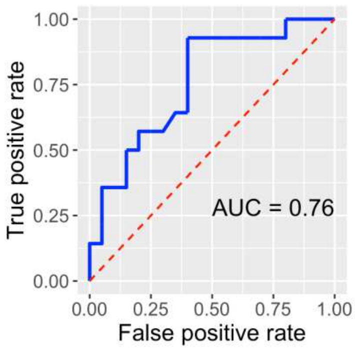
</td></tr>
<tr><td>TMB
predicted by
model_115
(best model)</td><td>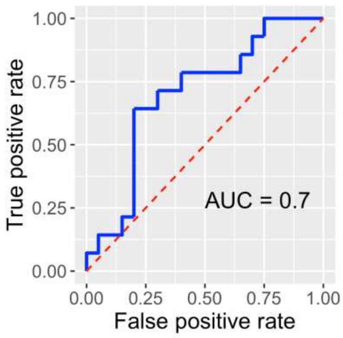
</td></tr>
<tr><td>TMB
predicted
by
model_73</td><td>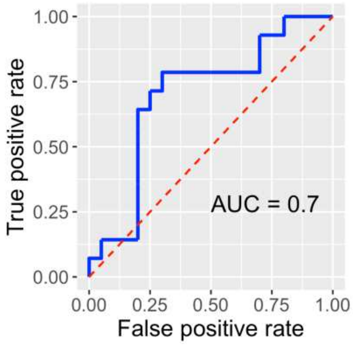
</td></tr>
<tr><td>TMB
predicted
by
model_37</td><td>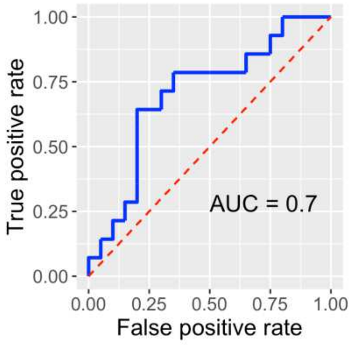
</td></tr>
</table>

Supplementary Figure 10: ROC curve of predicted TMB and type of response to ICB treatment in a cohort of 34 metastatic NSCLC patients

a

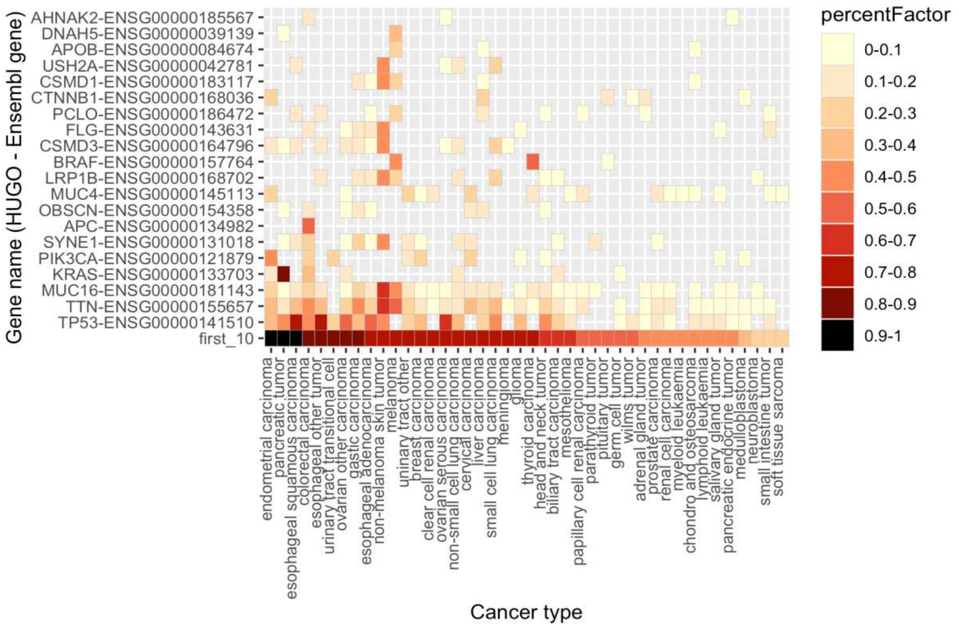

b

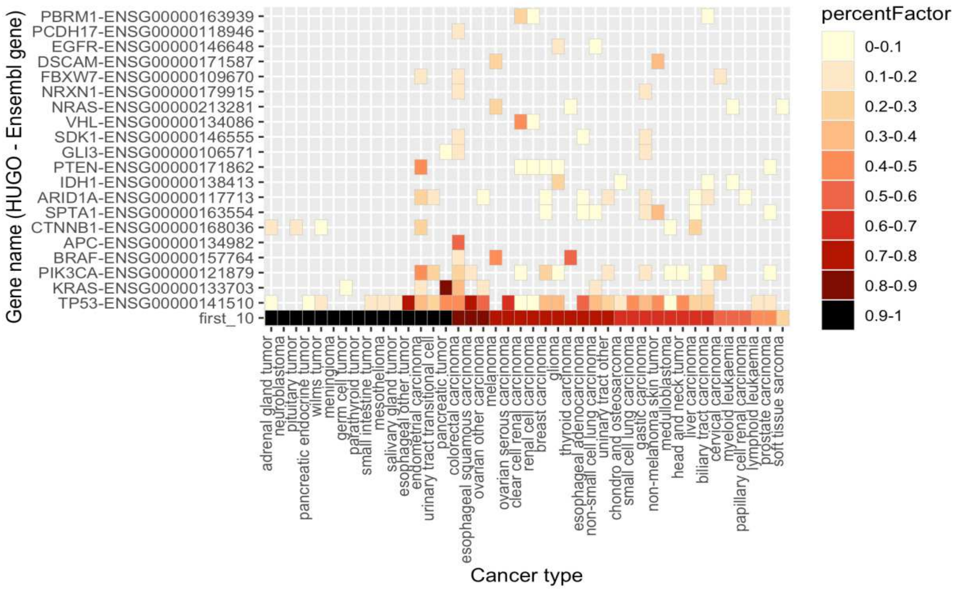

Supplementary Figure 11. Top mutant genes per cancer type before (a) and after penalization by length (b). The intensity of the colors indicates the percentage of mutant samples. To improve clarity, only 20 of the total 157 (**a**) and 195 (**b**) top 10 mutant genes are represented

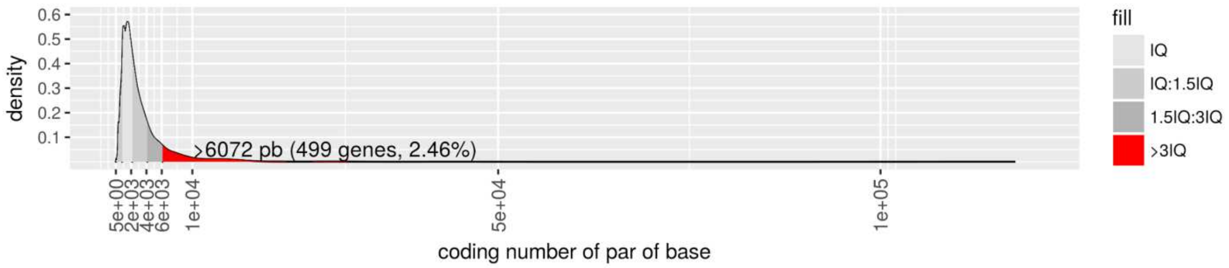

Supplementary Figure 12. Distribution of coding base-pairs among human genes (n= 20,291). 499 genes (2.46%) have coding regions longer than 3*IQ distance (in red)

A

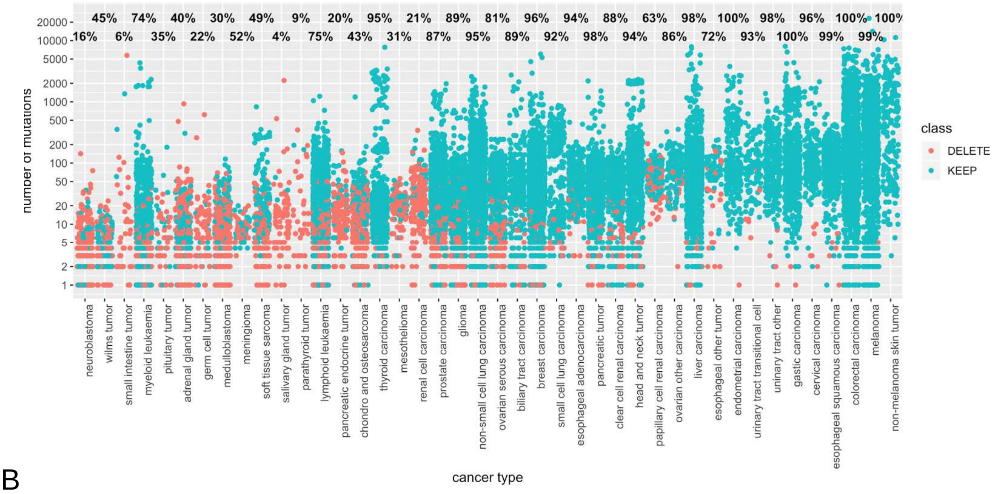

B

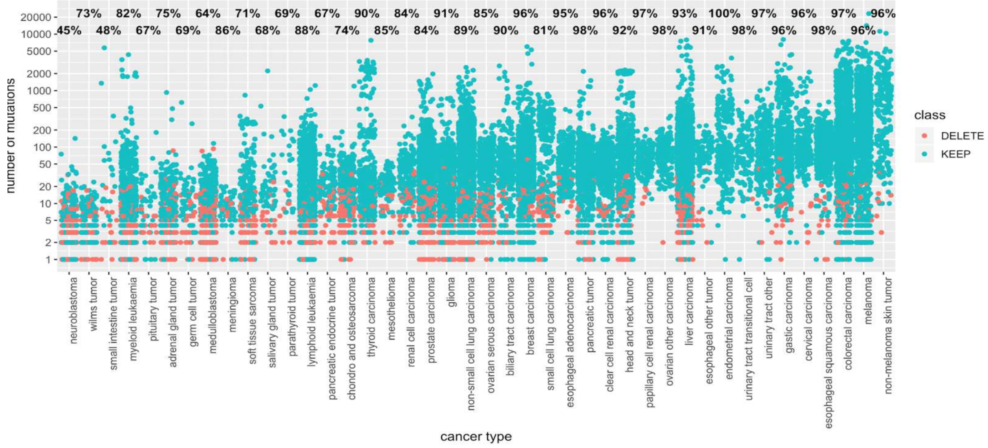

C:

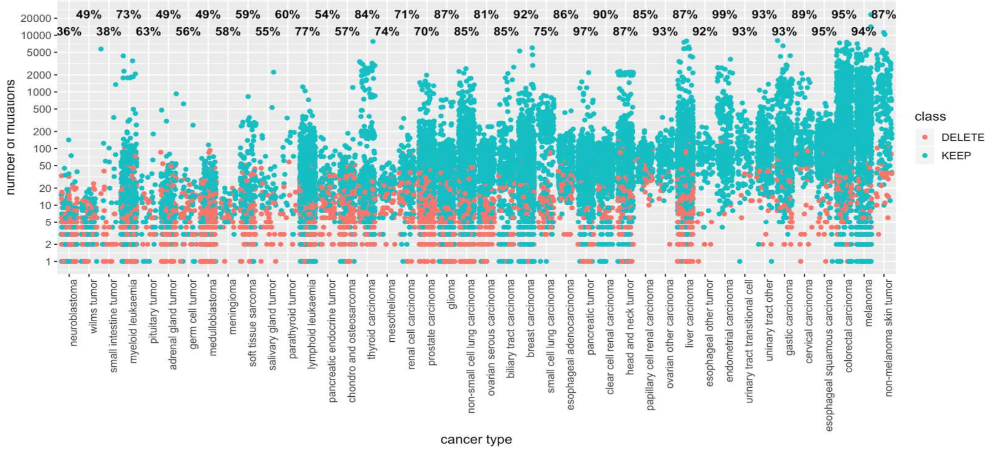

Supplementary Figure 13. Samples conserved and excluded in our analyses by cancer type and strategy: Each dot represents a conserved sample (blue) or a deleted sample (red). Numbers on top of each cancer type are the percentage of conserved samples. **A**: “our-exon” selection; **B**: CGC gene/exon selection; **C**: FO-panel gene/exon selection
