# Supplementary material for: Panels and models for accurate prediction of tumor mutation burden in tumor samples
Source: NPJ Precis Oncol. 2021 Apr 13;5:31. doi: 10.1038/s41698-021-00169-0 (PMC8044185; doi:10.1038/s41698-021-00169-0)
Supplement: Supplementary file 7 — Reporting Summary Checklist [file 41698_2021_169_MOESM7_ESM.pdf]

Corresponding author(s): Molina-Vila, MA; Marino-Buslje C

Reporting Summary

Nature Research wishes to improve the reproducibility of the work that we publish. This form provides structure for consistency and transparency in reporting. For further information on Nature Research policies, see Authors & Referees and the Editorial Policy Checklist.

Statistical parameters

When statistical analyses are reported, confirm that the following items are present in the relevant location (e.g. figure legend, table legend, main text, or Methods section).

n/a	Confirmed	
☐	☒	The exact sample size (*n*) for each experimental group/condition, given as a discrete number and unit of measurement
☐	☒	An indication of whether measurements were taken from distinct samples or whether the same sample was measured repeatedly
☐	☒	The statistical test(s) used AND whether they are one- or two-sided<br>*Only common tests should be described solely by name; describe more complex techniques in the Methods section.*
☐	☒	A description of all covariates tested
☐	☒	A description of any assumptions or corrections, such as tests of normality and adjustment for multiple comparisons
☐	☒	A full description of the statistics including central tendency (e.g. means) or other basic estimates (e.g. regression coefficient) AND variation (e.g. standard deviation) or associated estimates of uncertainty (e.g. confidence intervals)
☐	☒	For null hypothesis testing, the test statistic (e.g. *F*, *t*, *r*) with confidence intervals, effect sizes, degrees of freedom and *P* value noted<br>*Give P values as exact values whenever suitable.*
☒	☐	For Bayesian analysis, information on the choice of priors and Markov chain Monte Carlo settings
☒	☐	For hierarchical and complex designs, identification of the appropriate level for tests and full reporting of outcomes
☐	☒	Estimates of effect sizes (e.g. Cohen's *d*, Pearson's *r*), indicating how they were calculated
☐	☒	Clearly defined error bars<br>*State explicitly what error bars represent (e.g. SD, SE, CI)*

Our web collection on statistics for biologists may be useful.

Software and code

Policy information about availability of computer code

Data collection	As a training dataset, we downloaded the whole genome (WGS) and whole exon sequencing (WES) datasets of the Cosmic version 84 (Cosmic_v84) for human grch38 assembly, which contained 25,533 tumor samples. The datasets and codes generated and/or analyzed during the current study are available in https://gitlab.com/bioinformatics-fil/predict_tmb. For details about the software used, see supplementary methods.
Data analysis	All the programming and statistics were performed with R. The following R libraries were used; Genomic Features, biomaRt1 , GenomicRanges and rtracklayer. Translation databases hg18ToHg38.over.chain and hg19ToHg38.over.chain from UCSC web page (http://genome.ucsc.edu/) were employed for the filtering datasets process. All graphics were made with ggplot2. Library caret was used for internal and external validation. The datasets and codes generated and/or analyzed during the current study are available in https://gitlab.com/bioinformatics-fil/predict_tmb.

For manuscripts utilizing custom algorithms or software that are central to the research but not yet described in published literature, software must be made available to editors/reviewers upon request. We strongly encourage code deposition in a community repository (e.g. GitHub). See the Nature Research guidelines for submitting code & software for further information.

Data

Policy information about availability of data

All manuscripts must include a data availability statement. This statement should provide the following information, where applicable:

- Accession codes, unique identifiers, or web links for publicly available datasets
- A list of figures that have associated raw data
- A description of any restrictions on data availability

The data generated and analysed during this study are described in the following data record: https://doi.org/10.6084/m9.figshare.14074451. The datasets and code are available as part of this data record, and also via https://gitlab.com/bioinformatics-fil/predict_tmb. A list of the files underlying the figures, tables and supplementary tables of the related manuscript is available as part of the data record in the file 'Martinez-Perez_et_al_underlying_data_list.xlsx'. For details about the software used, see the methods of the related publication

Field-specific reporting

Please select the best fit for your research. If you are not sure, read the appropriate sections before making your selection.

☒ Life sciences ☐ Behavioural & social sciences ☐ Ecological, evolutionary & environmental sciences

For a reference copy of the document with all sections, see nature.com/authors/policies/ReportingSummary-flat.pdf

Life sciences study design

All studies must disclose on these points even when the disclosure is negative.

Sample size	Training dataset: We downloaded the whole genome (WGS) and whole exon sequencing (WES) datasets of the Cosmic version 84 (Cosmic_v84) (39) for human grch38 assembly, which contained 25,533 tumor samples classified into cancer types according to primary site, primary histology and histology subtype. Then, we excluded (i) benign tumors, (ii) samples with mutations without annotation of genomic coordinates, (iii) samples with non somatic mutations (labeled as "Variant of unknown origin" or "Not specified"), (iv) samples with mutations exclusively in non coding regions. Finally, cancer types with less than 10 samples were also excluded. Our training dataset contained 24,726 samples of 42 cancer types
Data exclusions	We excluded (i) benign tumors, (ii) samples with mutations without annotation of genomic coordinates, (iii) samples with non somatic mutations (labeled as "Variant of unknown origin" or "Not specified"), (iv) samples with mutations exclusively in non coding regions.
Replication	The external validation dataset was based on the new WGS and WES samples added to COSMIC_v90 (n=3144), together with samples with WGS and WES data retrieved from 133 articles publicly available in the literature (n=4773). All samples were mapped to the grch38 genomic coordinate and filtered according to the procedure described for the training dataset. Overall, the external validation dataset contained 7917 samples from 40 cancer types.
Randomization	n/a
Blinding	n/a

Reporting for specific materials, systems and methods

Materials & experimental systems

n/a	Involved in the study
☒	☐ Unique biological materials
☒	☐ Antibodies
☒	☐ Eukaryotic cell lines
☒	☐ Palaeontology
☒	☐ Animals and other organisms
☒	☐ Human research participants

Methods

n/a	Involved in the study
☒	☐ ChIP-seq
☒	☐ Flow cytometry
☒	☐ MRI-based neuroimaging
